# Molecular Basis of Encapsidation of Hepatitis C Virus Genome

**DOI:** 10.3389/fmicb.2018.00396

**Published:** 2018-03-07

**Authors:** Guoli Shi, Tetsuro Suzuki

**Affiliations:** ^1^Antiviral Immunity and Resistance Section, HIV Dynamics and Replication Program, Center for Cancer Research, National Cancer Institute, Frederick, MD, United States; ^2^Department of Virology and Parasitology, Hamamatsu University School of Medicine, Hamamatsu, Japan

**Keywords:** hepatitis C virus, encapsidation, packaging signal, *cis*-acting element, virion assembly

## Abstract

Hepatitis C virus (HCV), a major etiologic agent of human liver diseases, is a positive-sense single-stranded RNA virus and is classified in the *Flaviviridae* family. Although research findings for the assembly of HCV particles are accumulating due to development of HCV cell culture system, the mechanism(s) by which the HCV genome becomes encapsidated remains largely unclear. In general, viral RNA represents only a small fraction of the RNA molecules in the cells infected with RNA viruses, but the viral genomic RNA is considered to selectively packaged into virions. It was recently demonstrated that HCV RNAs containing 3′ end of the genome are selectively incorporated into virus particles during the assembly process and the 3′ untranslated region functions as a *cis*-acting element for RNA packaging. Here, we discuss the molecular basis of RNA encapsidation of HCV and classical flaviviruses, contrast with the packaging mechanism of HIV-1.

## Introduction

Hepatitis C virus (HCV) infection is a major cause of chronic hepatitis, liver cirrhosis, and hepatocellular carcinoma worldwide. In spite of the fact that HCV is targeted by innate, cellular and humoral immune mechanisms, its long-standing persistent infection can be established in a majority of the infected individuals. The recent development of direct-acting antivirals, which target specifically HCV non-structural proteins, initiated the era of high efficacy and well-tolerated medications with high cure rates (Bourlière et al., 2017).

Hepatitis C virus is classified in the *Hepacivirus* genes within the *Flaviviridae* family, which includes the classical flaviviruses such as yellow fever virus and dengue virus. The viral particle consists of a nucleocapsid, surrounded by a lipid envelope containing two viral glycoproteins, E1 and E2 (Moriishi and Matsuura, 2016). A hallmark of HCV particles is their association with cellular lipoproteins that potentially determine both morphology and biophysical properties of the virion (Suzuki, 2017). Although significant progress has been made regarding molecular biology of HCV life cycle over the last decade, our understanding of mechanisms on virion assembly, in particular, encapsidation of the viral genome has been limited.

In this short review, we summarize our current knowledge of moleculer basis of packaging of genomic RNAs of HCV and the classical flaviviruses. We also provide an overview of the mechanism on selective packaging of the HIV-1 genome and compare it with the HCV encapsidation.

## Genome Structure of HCV

The genome of HCV is a positive-sense single-stranded RNA with highly structured elements, which is about 9.6 kb in length (Choo et al., 1989). This genomic RNA contains one single open reading frame (ORF) encoding a polyprotein which can be processed into 10 viral proteins after translation. The HCV genome was flanked by the 5′ untranslated- (5′ UTR) and the 3′ untranslated- (3′ UTR) regions at the 5′- and 3′ ends, respectively. Both UTRs are highly structured and crucial for the viral translation and proliferation and are well conserved among genotypes or strains of HCV.

**FIGURE 1:**
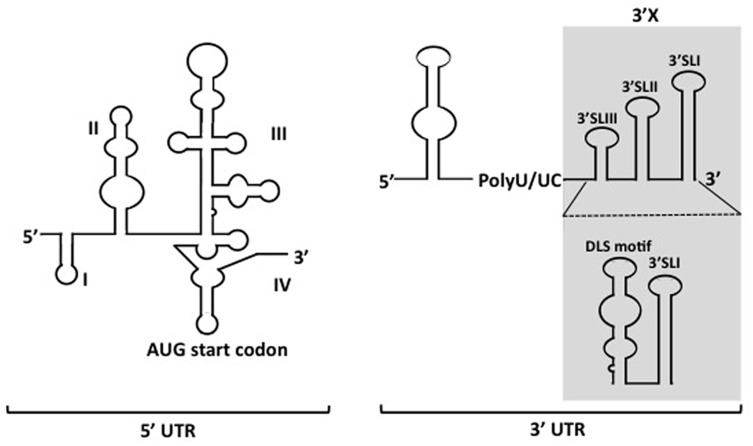
Depicted secondary structures of 5′- and 3′ UTRs of HCV genome. The 5′ UTR contains four structured domains (I–IV). The translational start codon is indicated. Two types of conformations for the 3′X region (*shadowed region*) within the 3′ UTR have been proposed.

The 5′ UTR is a ∼340-nucleotide (nt) element composed by four highly structured domains and is implicated in almost the whole processing of HCV life cycle, except virions entry. Domain I of the 5′ UTR comprises a single stem-loop. Domains II to IV of the 5′ UTR constitute an internal ribosomal entry site (IRES), which is a prerequisite for cap-independent translation of viral RNA (**Figure 1**) (Wang et al., 1995; Honda et al., 1996). While in general miRNAs interact with the 3′ UTR of mRNAs to promote mRNA destabilization and/or translational repression, the miR-122 binding to the 5′ UTR of HCV RNA is essential for the viral replication (Jopling, 2008; Machlin et al., 2011). The 3′ UTR varies between 200 and 235 nt in length, including a short variable region, as well as a poly(U/UC) stretch with a length of about 90 nt, and a virtually invariant 98-nt X-tail region (3′X). The 3′ UTR is vital for HCV genome replication. Several deletions or substitution mutations within the region resulted in loss of the viral replication (Friebe and Bartenschlager, 2002). Although the IRES of 5′ UTR is sufficient to initiate translation of mRNA in reporter systems, it was suggested that its translation efficiency can be elevated in the presence of 3′ UTR, possibly through stabilizing the RNA and forming the RNA complex with 5′ UTR (Song et al., 2006; Bai et al., 2013). However, no or only a limited contribution of 3′ UTR to stabilization of the viral genome was observed in our trans-packaging system (Shi et al., 2016). The 3′X tail has been described to fold into two conformations, one of which is composed with three stem loops, while the other consists of two stem loops. In both predicted conformations, the SL I at the very end of 3 terminus is preserved, while the upstream 55 nt-long segment forms either a single stem loop exposing the dimer linkage sequence (DLS) or two stem loops (SLII and SLIII) (**Figure 1**) (Ivanyi-Nagy et al., 2006; Shetty et al., 2010; Palau et al., 2013; Romero-López et al., 2014). Though DLS was found inevitable to form HCV RNA homodimers *in vitro*, it is not yet clear whether homodimers are formed in infected cells. Since involvement of the DLS region in HCV RNA dimerization would preclude its ability to engage in the long-range kissing interaction with the NS5B-coding region that is known to be essential for RNA replication, and the HCV virion contains a single copy of the genomic RNA, DLS might be an intermediate conformation during switching between processes of genome replication and/or encapsidation. The 3′ tail often forms long distance complexes with RNA structures, including elements within the NS5B coding sequence in HCV genome. Among the structured RNA elements in the coding region, the *cis* replication element (CRE) located at NS5B region, which consists of three conserved domains, 5BSL3.1, 5BSL3.2, and 5BSL3.3, have been demonstrated for their regulatory roles in translation and genome replication via long distance interacting with 5′ UTR, 3′ UTR and SL9033 (a structured RNA element upstream of CRE) (You et al., 2004; Friebe et al., 2005; Diviney et al., 2008; Romero-Lopez and Berzal-Herranz, 2009; Shetty et al., 2010; Tuplin et al., 2012). The polyU/UC stretch provides quite a spatial flexibility to the stem-loops in 3′ UTR, allowing spatial contact with CRE. Thus, the length of the polyU/UC stretch as well as UTP contents in the region are important for the viral replication (You and Rice, 2008).

## Mechanistic Analysis of Encapsidation of the HCV Genome

Although evidence regarding the viral replication and particle formation of HCV are accumulating due to development of HCV cell culture system, detailed mechanisms for incorporation of the viral genome into progeny virions has remained largely unclear. Very few evidence on HCV RNA elements contributing to the viral genome packaging has been scattered. An early study demonstrated that the 5′ UTR of the genome most likely does not contain *cis*-acting RNA structures required for the viral encapsidation (Friebe and Bartenschlager, 2009). In our recent study, the HCV 3′ UTR was found to function as a *cis*-acting element, which is important in viral genome packaging, as described below (Shi et al., 2016).

In the livers and sera of patients with HCV infection, in addition to HCV full genome RNA, a large portion of 5′-end subgenomic HCV RNAs were found, which were possibly generated by premature transcription termination or RNA cleavage at particular sites within the viral genome (Quadri and Negro, 2001; Shimizu et al., 2006). Accordingly, in the HCV cell culture system, we also found greater distribution of 5′-end subgenomic RNAs over the intact HCV genome. By quantitating HCV RNAs both at 5′ end and 3′ end sites, we found that the 3′/5′-end ratio of the viral RNA copies in culture supernatants of HCV-infected cells was markedly higher than that in the infected cells, confirming over-distribution of 5′ end subgenomic HCV RNAs in the viral replicating cells. Further higher 3′/5′ ratios were observed in the density fractions with high infectivity rather than the whole culture supernatants, indicating a positive correlation between 3′/5′ ratio of the HCV RNA copy and the viral infectivity. Further experiments to comprehensively understand the encapsidation of HCV genome demonstrated that the size of most of RNA species extracted from purified HCV particles appears to correspond to the full-length of the viral genome by judged by the bioanalyzer electrophoresis (unpublished data). Thus, it is likely that the HCV RNA species with the genomic-size or nearly so are positively selected and incorporated into the viral particles during the encapsidation processing.

Regarding to currently revealed mechanisms of virus encapsidation, which are generally initiated via recognition of the structured element(s) within genomic nucleic acids, termed packaging signal(s), by the viral capsid or coat protein, we hypothesized that the specific packaging of HCV genome is driven by interaction of the viral RNA with capsid protein, Core. Our *in vitro* binding assays showed that amongst the structured HCV RNA elements tested, 3′ UTR possessed higher Core-binding affinity compared to other RNA elements such as 5′ UTR and the elements in NS5B coding region and that their affinity was independent of the lengths of RNA sequences (Shi et al., 2016). Notably, the RNA fragment with both 3′ UTR and CRE showed lower Core-binding affinity than that with 3′ UTR alone, speculating that the long-distance kissing-loop structure consisting of CRE and stem-loop(s) within 3′ UTR led to masking certain motif(s) involved in the Core binding. Together with the finding that the kissing-loop interaction of the HCV genome is important for RNA replication, it is reasonable to consider that conformational alteration of the 3′-end regions of the HCV genome may contribute to switching the process during HCV life cycle from RNA replication to the early phase of particle formation.

**FIGURE 2:**
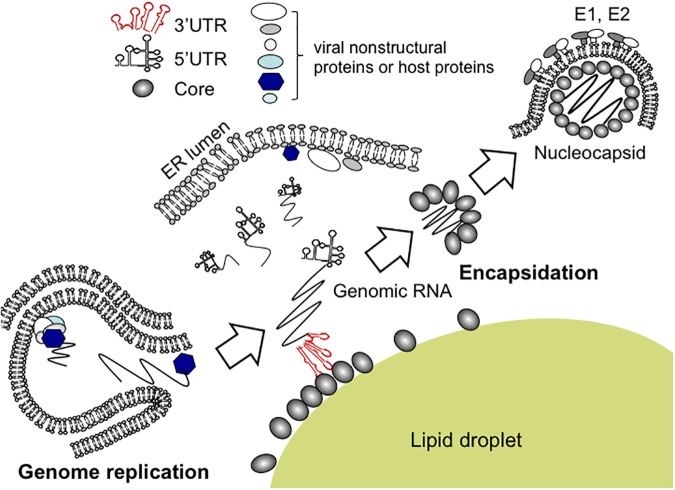
Proposed model for encapsidation of HCV genome. Newly synthesized HCV genomic RNA is released from membrane vesicles containing the replication complex (*lower left*). It may be likely that the viral genome bound to NS5A is recruited to the surface of lipid droplets, enabling its interaction with Core. For nucleocapsid formation, the 3′ UTR of the HCV genome acts as a *cis*-acting element for RNA packaging. Within the 3′ UTR region, the loop sequences of stem-loop structures appear to be essential for HCV encapsidation.

Although findings from the *in vitro* binding assays suggested that 3′ UTR, rather than other RNA elements tested, is potentially implicated in the viral encapsidation, only successful directing of RNA into virions could authentically prove its role as a *cis*-packaging signal. By utilizing a *trans*-packaging system (Masaki et al., 2010) with replication-defective HCV subgenome, we found that deletion of 3′ UTR is deleterious for production of trans-complemented HCV particles (HCVtcp) while deletion of 5′ UTR also impaired it to some extent (Shi et al., 2016). RNA stability of deletion mutants used was confirmed to be comparable to that of intact subgenomes. Notably, the loop motifs of 3′X tails in 3′ UTR were found to be important for binding to Core as well as HCVtcp production. Substitutions in the loop sequences of which secondary structures were not affected, resulted in marked impairment of the Core-binding and RNA packaging into the viral particles (Shi et al., 2016). Stewart et al. used *in vitro* systematic evolution of ligands by exponential enrichment (SELEX) to screen RNA aptamers that bind specifically to Core and identified 8 short RNA motifs within the HCV ORF that are possibly involved in the virion assembly or the late stages of the HCV life cycle (Stewart et al., 2016). Aptamers within a randomized library possess their unique tertiary structures, and are present in their most stable conformations. SELEX is therefore likely to enrich for aptamers with conserved conformation rather than a unique primary sequence. It is possible that multiple RNA elements are involved in genome packaging of HCV, in addition to 3′ UTR and 5′ UTR, at the physiological environment. While the motifs scattered within genome may assist the encapsidation through interacting with Core, the viral 3′ UTR may also play a role in recruiting cellular and/or other viral factors that are important for genome packaging or virus assembly (Harris et al., 2006). RNA aptamers selected against HCV NS3 helicase were found to share high similarities with 3′ UTR (Nishikawa et al., 2004). It may be possible that 3′ UTR undergoes conformational alteration mediated by NS3 helicase during replication events and/or at switching from replication to encapsidation. As a capsid protein, HCV Core may have an ability to facilitate the inter-conversion between diverse RNA structures, leading to regulating structural transitions of the viral RNA elements during HCV life cycle (Cristofari et al., 2004; Gawlik and Gallay, 2014). 3′ UTR has been found to activate an IKK-α-dependent pathway that induces lipogenic genes and to enhance Core-associated lipid droplet formation beneficial to the viral assembly (Li et al., 2013). Indeed, in the *trans*-packaging system 3′ UTR appeared to facilitate the interaction between Core and NS5A at or around lipid droplets. NS5A, a phosphorylated non-structural protein of HCV, is a multi-functional protein required for RNA replication and virion assembly (Masaki and Suzuki, 2015). The Core-NS5A interaction is thought to be crucial for the early phase of the particle formation (Appel et al., 2008; Masaki et al., 2008). A proposed model of encapsidation of HCV genome is illustrated in **Figure 2**.

## The RNA Structures in Genomes of Flaviviruses and Potential Molecular Basis of Encapsidation

The *Flaviviridae* is a family of positive, single-stranded enveloped RNA viruses and the family has four genera; *Flavivirus*, *Hepacivirus* (type species HCV), *Pegivirus* and *Pestivirus*. Flaviviruses, members of genus Flavivirus, such as the yellow fever virus and dengue virus, contains a range of zoonotic viruses and are largely transmitted to vertebrate hosts by arthropod vectors. The genomic organization of flaviviruses shared great similarity with HCV, and the genome consists of a single large open reading frame flanked by a short 5′ UTR and a short 3′ UTR (Chambers et al., 1990). Different from HCV, the genomic RNA is modified at the 5′ end of positive-strand genomic RNA with a cap-1 structure (me^7^-GpppA-me^2^), thus translation of the flavivirus genome is Cap-dependent. There are two conserved structural elements in 5′ UTR, a large stem loop (SLA) and a short stem loop (SLB). SLA is likely to act as a promoter, which is essential for viral RNA synthesis. SLB is involved in interactions between the 5′ UTR and 3′ UTR. The 3′ UTRs are typically 0.3–0.5 kb in length and contain several highly conserved structures, some of which have been characterized to be important in facilitating the viral genome replication (Clarke et al., 2015; Fernández-Sanlés et al., 2017). The interactions between 5′ UTR and 3′ UTR of the flavivirus genome result in the cyclisation of the viral RNA, which is essential for viral replication (Alvarez et al., 2005). However, packaging signals or *cis*-acting elements important for RNA packaging have not been identified in the genomic RNA thus far. It has been considered that replication and encapsidation of flaviviruses are functionally coupled, which was evidenced by unable to detect infectious virus particles production with a plasmid DNA encoding replication-deficient full-length Kunji virus cDNA (Khromykh et al., 2001b). As it happens with HCV, HCVtcp production in the *trans*-packaging system with replication-defective subgenome was approximately 10-fold lower compared to that with the replication-competent setting (Shi et al., 2016). One could argue that the active replication machineries of flaviviruses and HCV not only provide continuous transcription and accumulation of genomic RNA, but also potentially recruit factors that are important for efficient encapsidation of genomic RNA. It has been described that direct interaction between 5′ and 3′ terminal nucleotide sequences of flaviviruses genome RNA mediated the virus RNA cyclization (Khromykh et al., 2001a). The significance of genome cyclization during virus life cycle has been uncovering. Evidences demonstrated that genome cyclization is implicated in regulating genome replication (Mir et al., 2006; Villordo and Gamarnik, 2009). It is still to be explored whether the cyclization is involved in flaviviruses encapsidation. This long-range RNA-RNA interaction could also serve as a molecular switch during different stage of virus replication, similarly as in HCV replication.

## Enlightenment From Studies of Genome Packaging of Retroviruses

So far, the packaging of genomic RNA of retroviruses has been better elucidated than any other RNA viruses. Herein, we will take HIV-1 as an example to briefly compare the packaging processing with that of HCV. Upon viral entry to the target cells, the RNA genome of HIV is reverse-transcribed into double-stranded DNA, followed by integrating into the cellular genome to form a provirus (Freed, 2015). The spliced- and unspliced viral transcripts are exported from nuclear to cytoplasm for subsequent processing. Genome dimerization is vital for HIV packaging. upon dimerization, a pair of unspliced genomic RNAs were packaged into virions (Skripkin et al., 1994; Russell et al., 2003; Paillart et al., 2004). To accomplish the specific packaging of the viral genome, HIV-1 uses the *cis*-acting RNA elements in the viral genome and the *trans*-acting elements in Gag protein (D’Souza and Summers, 2005). The 5′ UTR contains the packaging signal of HIV-1, psi (Ψ) (Lever et al., 1989), which consists of a series of stem-loops, as well as a short sequence required for the initiation of RNA dimerization (McBride and Panganiban, 1996; Lu et al., 2011). A part of the 5′ end of Gag-coding sequence is also involved in the encapsidation (Chamanian et al., 2013; Olson et al., 2015; Comas-Garcia et al., 2016). Packaging of HIV genome is initiated by the recognition of packaging signals, which is a prerequisite for assembly of HIV particles with full-length genomic RNA. It was recently unveiled that binding of Gag at the cell membrane stabilized the dimerization of two copies of the genomic RNA (Chen et al., 2016), thus paved the way for assembly particles and production of progeny viruses. The generality of the encapsidation mechanism between HIV-1 and HCV is that both use highly conserved and structured RNA elements for recognition by the viral coat protein and that, to achieve maximum packaging efficiency, multiple RNA elements within the viral genome play cooperatively while with one element as dominating packaging signal. The HIV-1 RNA packaging signal is recognized by Gag, which is synthesized as a polyprotein that is later processed into six mature proteins: matrix, capsid, SP1, nucleocapsid (NC), SP2, and p6. NC plays the leading role for the selective packaging of the HIV-1 RNA genome (Bell and Lever, 2013). Mutations of the zinc knuckle motifs in NC can cause severe defects in the viral RNA packaging (Guo et al., 2002). A recent study reported that a Gag mutant in which four basic residues between the two zinc fingers have been replaced with alanines retained significant affinity for the packaging signal, but not control RNA (Comas-Garcia et al., 2017), suggesting that these four basic residues help to maintain the proper conformation of the NC domain as well as contributing to the electrostatic interactions with RNA (Webb et al., 2013). Intriguingly, we also found that the basic residues in N-terminus of HCV Core are critical for both specific interaction with HCV RNA and electrostatic interactions (unpublished data).

## Future Perspectives

There is accumulating evidence regarding nucleic acids packaged in the virus particles determined by new-generation sequencing techniques. In addition to the viral RNAs, significant amounts of host RNAs are detectable in HIV-1 particles, such as host mRNAs, tRNAs, and 7SL RNA (Telesnitsky and Wolin, 2016). Recruitment of these RNAs into HIV particles is considered to be independent of the viral genome packaging and to be mediated by the viral and/or host RNA-binding proteins through less sequence-specific interactions. In our primary analysis, host-derived RNA species were also found in HCV particles (unpublished data). How these host-cell RNAs are packaged into HCV virus particles as well as the virological significance of non-viral nucleic acids present in the virus particles remains to be elucidated.

For several positive-strand RNA viruses; not only flavivirus (Khromykh et al., 2001b), but poliovirus (Nugent et al., 1999) and bromovirus (Annamalai and Rao, 2006), functionally coupling of RNA packaging and replication has been reported. It is likely that a replication-coupled packaging mechanism potentially permits efficient access of the capsid protein to interact with progeny viral RNAs. Functional active replication machinery might also recruit factors promoting encapsidation efficiently at the site for nucleocapsid formation. However, this coupling mechanism might result in competing between capsid protein and the replicase for RNA binding. To advance our understanding of the encapsidation of positive-strand RNA viruses, further researches on their regulatory mechanisms for the link between the RNA replication cycle and virus assembly are required. Specific recognition of the genomic RNA via the packaging signal and coupling with replication are not mutually exclusive, but seem rather cooperate for the best beneficial of encapsidation and nuclecapsid formation. Our methodology in investigating HCV encapsidation could be applied to explore the fundamental *cis*-acting RNA elements that are crucial for the genome packaging of flaviviruses or other positive-strand RNA viruses.

## Author Contributions

All authors listed have made a substantial, direct and intellectual contribution to the work, and approved it for publication.

## Conflict of Interest Statement

The authors declare that the research was conducted in the absence of any commercial or financial relationships that could be construed as a potential conflict of interest.
